# Optimization of Osmotic Dehydration of Autumn Olive Berries Using Response Surface Methodology

**DOI:** 10.3390/foods10051075

**Published:** 2021-05-13

**Authors:** Mohamed Ghellam, Oscar Zannou, Hojjat Pashazadeh, Charis M. Galanakis, Turki M. S. Aldawoud, Salam A. Ibrahim, Ilkay Koca

**Affiliations:** 1 Food Engineering Department, Faculty of Engineering, Ondokuz Mayis University, Samsun 55000, Turkey; zannouoscar@gmail.com (O.Z.); hojjat_pashazadeh@yahoo.com (H.P.); itosun@omu.edu.tr (I.K.); 2 Research & Innovation Department, Galanakis Laboratories, 73100 Chania, Greece; cgalanakis@chemlab.gr (C.M.G.); tdawoud@ksu.edu.sa (T.M.S.A.); 3 Food Waste Recovery Group, ISEKI Food Association, 1190 Vienna, Austria; 4 Department of Botany & Microbiology, College of Science, King Saud University, Riyadh 11451, Saudi Arabia; 5 Food and Nutritional Sciences Program, North Carolina A&T State University, Greensboro, NC 27411, USA; ibrah001@ncat.edu

**Keywords:** autumn olive, osmotic dehydration, optimization, response surface methodology

## Abstract

Autumn olive fruits are a rich source of nutrients and functional compounds, making them functional foods against many diseases and cancers. To increase the consumption, its processing, and its transformation into new products would help spread them to the consumer’s table. In this study, after giving an overview of the physicochemical characteristics and the antioxidant activity, the objective was to optimize the osmotic dehydration (OD) of the berries. Response surface methodology was used to investigate the effect of dehydration factors: syrup concentration (30–70%), temperature (20–70 °C), and fruit-to-syrup ratio (1:10–2:10) on the water loss (WL), sugar gain (SG), weight reduction (WR), density (ρ), water activity (a_w_), and total color change (ΔE) of fruits after 10 h of OD. Results obtained by employing Box–Behnken design (three variables, three levels), and significant terms of regression equations indicated that the syrup concentration and temperature variation are the most affecting factors on the previously mentioned independent variables (WL SG, WR, ρ, a_w_, and ΔE). Fruits to syrup ratio appeared to have a significant effect only on WL. Under the optimum conditions found (70%, 70 °C, 1.8:10), the predicted values were 59.21%. 19.21%, 32.34%, 1.22 g/cm^3^, 0.850, and 3.65 for WL, SG, WR, ρ, a_w_, and ΔE, respectively.

## 1. Introduction

The role of food bioactives in protecting consumers’ health has been upgraded in the era of the COVID-19 pandemic [1,2]. Food bioactives are mainly found in fruits and vegetables [3]. For instance, autumn olive fruit (*Elaeagnus umbellata*) is a delicious reddish berry from the Elaeagnaceae family that comprises a good source of various nutrients and antioxidant compounds. These berries can contain more than 15 times more lycopene than tomatoes. Therefore, autumn olive berries can play a useful role in protecting consumers’ health and reducing the risk of contracting many diseases [4,5]. These miraculous berries show great industrial potentials since they can be used and processed in food industries [6] and in pharmaceutical and cosmetic industries.

Autumn olive fruits are perishable within only a few days due to the high water content and high fragility of the fruit skin. Thus, these berries are vulnerable and need more attention during the harvest and postharvest processing. In order to increase their shelf life and decrease their vulnerability, these fruits can be subjected to many transformation or treatment techniques. Since water is the dominant component in fruits and vegetables, it plays a critical role in influencing foodstuffs’ quality and stability due to the possible series of physicochemical and biological changes. Drying is one of the essential preservation techniques used to increase storage stability and reduce the packaging and transport costs, making commodities affordable in their off-seasons. This technique has been widely used for centuries [7,8].

Osmotic dehydration (OD) can be used as a pre-treatment for a large number of foodstuffs (fruits, vegetables, meat, seafood, etc.) before performing the primary preservation process. It consists of reducing the initial water content [9]. Generally, the osmotic process appears to be a vital technique since it can preserve and improve the nutritional, organoleptic, and overall functional properties of osmosed products [10,11]. Osmotic treatment is a desirable process because of its effectiveness in preventing and minimizing discoloration and the loss of flavor, attributed to slight thermal damage (low temperatures) and delayed enzymatic browning reactions [12,13]. Furthermore, OD is, among drying technologies, described as a tool that requires less energy consumption and operational costs [8,9].

OD is generally defined as the partial removal of water from cellular materials by immersion in a hypertonic solution. The driving force applied by osmotic pressure triggers a mass transfer between the two media: a diffusion of water from the tissues into the osmotic solution, and, simultaneously, a counter-diffusion of solute from the solution into the cellular tissues. The diffusion takes place throughout the semi-permeable cell membrane. Therefore, OD is not only a water removal treatment but also a significant solute incorporation into food. Nonetheless, OD’s application leads to a weight reduction of the osmotically treated product [9,13].

Various products can be made through OD processes, including mainly candied and dried fruits and vegetables. These foods can be consumed directly or added as ingredients in many traditional and novel food products and preparations, including ice cream, bread, and cakes. OD can be followed by complementary treatment to continue the preservation process, such as drying (air/vacuum/freezing), freezing, canning, etc. Furthermore, this pre-treatment process has been recently combined with numerous techniques such as vacuum, centrifugal force, and high-intensity ultrasound. The applications of these treatments have greatly improved the mass transfer (solid gain and water loss) and the subsequent drying properties (rate and time), and hence, the final product quality [14,15,16].

Fruits and vegetables can be osmosed whole, in pieces, or in different sizes and shapes. Many osmotic media are applied as concentrated solutions (e.g., sucrose, sugar, corn syrup, salt). Several process parameters have been found to be critical for the mass transfer, such as the osmotic solution concentration, treatment temperature, solute properties, sample to solution ratio, agitation, time, pressure (vacuum, high pressure), and food characteristics (e.g., shape, size, structure, tissue compactness) [17].

At the start of this work, the physicochemical properties and antioxidant activity of autumn berries were determined to give a general overview of this nutrient-rich source and its potential activity. To the best of the authors’ knowledge, no research has been published on the OD of autumn berries. It is believed that the dried autumn berries would find a large market and receive a similar appeal from consumers like other dried berries (blueberry, blackberry, and cranberries), grapes, and other fruits. Therefore, this study aimed to investigate the dehydration of autumn olive berries by OD as a function of syrup concentration, osmotic temperature, and sample to solution ratio. For this purpose, the response surface methodology was used to determine the optimum conditions for OD.

## 2. Materials and Methods

### 2.1. Material and Chemical

Autumn olive fruits were harvested from the Department of Horticulture’s trial garden, Ondokuz Mayis University, Samsun, Turkey. Fruits were sorted, and the fruits with similar maturity (ripe red fruits) and shape were kept. Fruits were put into in refrigerator bags (ca. 300 g) and immediately frozen at −20 °C.

2,4,6-Tris(2-pyridyl)-1,3,5-triazine (TPTZ), 2,2-Diphenyl-1-Picrylhydrazyl (DPPH), 6-hydroxy 2,5,7,8-tetramethylchroman-2-carboxylic acid (Trolox), 2,6-dichlorophenolindophenol, hydrochloric acid (HCl, 37%), methanol (99.8%), acetone, oxalic acid, sodium nitrite, sodium hydroxide (NaOH), and (-)-epicatechin (Ep) were purchased from Sigma-Aldrich (St. Louis, MO, USA). Gallic acid (GA) and sodium carbonate from Riedel-de Haen (Seelze, Germany). Potassium chloride, sodium acetate, glacial acetic acid, and ascorbic acid (AA) from CARLO ERBA (Rodano, Italy). Aluminum chloride, Folin–Ciocalteau, iron chloride (Merck). Hexane (TEKKİM), BHT (butylated hydroxytoluene) (SAFC). Analytical balance (Radwag, AS 220/C/2, Radom, Poland), precision balance (Radwag, PS 3500.R1, Poland), hotplate stirrer (M TOPS, Multi-position), drying oven (NÜVE, FN 500P, Ankara, Turkey), shaking water bath (Model ST 30, NÜVE, Ankara, Turkey).

### 2.2. Physico-Chemical Analyses

The frozen fruits were sorted again from the damaged or crushed fruits, homogenized, and left to thaw at room temperature for the various analyses and the osmotic treatment. Afterward, the fruits were gently blotted with paper to remove the superficial humidity before being transferred.

pH was measured using a pH-meter (Model Starter 3100, OHAUS, Parsippany, NJ, USA). Titratable acidity (TA) was assessed and expressed as a malic acid concentration (%) [18]. Length and width were measured by a digital caliper (TRESNA, Series: EC16, China). Lycopene and vitamin C were determined according to Fish, Perkins-Veazie, and Collins [19]. The amount of total phenolic compounds (TPC) was measured spectrophotometrically at 760 nm according to the Folin–Ciocalteu method [20]. Total anthocyanin content was determined by the pH differential method [21]. Flavonoid content was determined using a spectrophotometric method based on the aluminum complex formation [22]. The free radical scavenging capacity was assessed by using Diphenyl-1-Picrylhydrazyl (DPPH) as a free radical [22]. The reducing capacity of berry extracts was measured according to the method of Benzie and Strain [23]. Minor modifications were performed for many analyses. Calibration curves were used to calculate concentrations; curves prepared by the corresponding standards (Trolox, GA, AA, Ep) showed high values of determination coefficient (R^2^ > 0.990). The previously mentioned analysis was performed for fresh berries with many replications.

For both the fresh and osmosed fruits, water activity (a_w_) was determined using a calibrated water activity meter at 25 ± 0.1 °C (Aqualab, 4TE, WA, USA). The bulk density (ρ) was measured according to Yang and Atallah [24]. Soluble solids contents (SSC) were analyzed by an Abbe refractometer (Model DTM-1, Atago, Tokyo, Japan). The fruits’ color was measured using a digital colorimeter (Model CR-400, Minolta-Konica Sensing Inc., Osaka, Japan). The CIE L*a*b* scale was used, and the total color change (ΔE) of the treated samples was calculated as with Equation (1):

(1) $$\Delta E\;=\;\sqrt{{\left(l-l_{0}\right)}^{2\;}+{\left(a-a_{0}\right)}^{2\;}+{\left(b-b_{0}\right)}^{2\;}}$$

where *l*_0_, *a*_0_, *b*_0_, and *l*, *a*, *b*, are the color parameters of fresh and treated fruits, respectively.

To determine moisture content, a homogenized quantity of the samples (ca. 5 g) was taken and dried at 70 °C till at a constant weight. Moisture content (M%) was calculated as follows:

(2) $$M\left(\%\right)=\frac{W_{i}-W_{f}}{W_{i}}\times 100$$

where W_i_ and W_f_ are the initial and final weights of the samples, respectively.

### 2.3. Water Loss, Solute Gain, and Weight Reduction

After the osmotic process, the mass transport of the fruits in sucrose solutions was expressed as water loss (WL), sugar gain (SG), and weight reduction (WR), and was determined according to the previous method [25]. The water loss was defined as the net loss of water from the autumn olive fruits (Equation (3)), the sugar gain as the net gain in total solids based on the initial mass (Equation (4)), and the weight reduction as the net mass reduction of the fruits based on the initial mass (Equation (5)).

(3) $$\mathrm{WL}=\frac{W_{i}X_{i}\;-W_{f}X_{f}}{W_{i}}\times 100$$

(4) $$\mathrm{SG}=\frac{W_{f}(1-X_{f})-W_{i}(1-X_{i\;})}{W_{i}}\times 100$$

(5) $$\mathrm{WR}=\frac{W_{i}-W_{f}}{W_{i}}\times 100$$

where WL is the water loss (g water/g initial mass of autumn fruits)%, SG is the sugar gain (g sugar/g initial mass of autumn fruits)%, WR is the mass reduction (g/g of the initial mass of autumn fruits)%, W_i_ is the initial mass of the autumn fruits (g), W_f_ is the mass of the autumn fruits after OD or at a specific time (g), X_i_ is the water content as a fraction of the initial mass of the autumn fruits, and X_f_ is the water content as a fraction of the mass of the fruits after OD.

### 2.4. Osmotic Dehydration (OD)

Osmotic solutions (syrups) at different concentrations (30–70%, *w*/*w*) were prepared with distilled water and commercial sugar purchased from the local market. The concentration of the solutions was controlled continuously by the Abbe refractometer before each use. The solutions were poured in 500 mL beakers placed on hot plates, and the temperatures (20–70 °C) were continuously controlled by a digital thermometer before and after adding the berries. The berries were added at different weight ratios to 400 g of solution (1:10–2:10). The beakers were tightly closed during the process to avoid water evaporation. The experimental time was fixed at 10 h for all experiments. Due to the low density of the fruits that floated on the surface and depending on the solution with the highest density (solution concentration), the agitation speed was fixed at 250 rpm for all runs. At the end of the process, the fruits were gently taken from the osmotic solutions and rinsed with distilled water (ca. 20 s). They were immediately blotted with paper to remove the excess surface water (2–3 min).

### 2.5. Scanning Electron Microscope (SEM)

Microstructures of the fresh and dried autumn olive berries were obtained by using SEM (JEOL JSM-7001F). A small specimen was cut from the samples and attached onto a stainless stub with double-sided sticky tape and sputtered immediately with a gold/palladium target (60/40) of approximately 10 nm using a sputter coater functioning with an argon and plasma current for 2 min. The images were observed at an acceleration voltage of 10 kV.

### 2.6. Experimental Design and Statistical Analysis

Response surface methodology (RSM) is an empirical statistical modeling technique employed for multiple regression analyses using quantitative data obtained from adequately designed experiments to simultaneously solve multivariate equations [26]. RSM was used to estimate the main effects of process variables on the water loss (WL), solid gain (SG), weight reduction (WR), water activity (a_w_), density (ρ), and color (ΔE) of the autumn berries. A Box–Behnken design was used for designing the experimental data. The independent variables were solution concentration (X_1_), temperature (X_2_), and fruit-to-solution ratio (X_3_). Using statistical software (Design-Expert 10, Minneapolis, MN, USA), the design generated 17 runs, including 5 center points. The following second-order polynomial equation was used to fit the experimental data:

(6) $$Y_{i}=\beta_{0}+\;\beta_{0}X_{1}\;+\;\beta_{2}X_{2}+\;\beta_{3}X_{3}+\;\beta_{12}X_{1}X_{2}+\;\beta_{13}X_{1}X_{3}+\;\beta_{23}X_{2}X_{3}+\;\beta_{11}X_{1}^{2}+\beta_{22}X_{2}^{2}+\beta_{33}X_{3}^{2}$$

where *Y_i_* is the response (WL, SG, WR, a_w_, ρ or ΔE), *β_n_* is the constant regression coefficients, the variables X_1_, X_2_, and X_3_ are the solution concentration (% *w*/*w*), temperature (°C), and fruit-to-solution ratio, respectively.

The statistical software Design-Expert 10 (Stat-Ease, Minneapolis, MN, USA) was used for statistical analysis, the generation of models, and the determination of optimum conditions and contours. Data were analyzed for the fitting of each model of the dependent variables (WL, SG, WR, a_w_, ρ, or ΔE) by multiple linear regression. The significance of the model (*p* < 0.05) was evaluated by analysis of variance (ANOVA) for each response. The adequacy of the models was checked in terms of a coefficient of determination (*R*^2^), adjusted coefficient of determination (Adj.*R*^2^), prediction error sum of squares (PRESS), and a lack-of-fit value. The optimum conditions were calculated according to the desirability function by maximizing WL, SG, WR, and ρ, minimizing a_w_, and ΔE, and keeping in range the independent variables. The non-significant factors were stepwise removed from the polynomial model.

## 3. Results and Discussion

### 3.1. Physico-Chemical Characteristic and Antioxidant Activity of Autumn Berries

In this work, the fresh, ripe berries of the autumn olive were analyzed to assess this fruit’s chemical and physical properties. This evaluation allows for a better understanding of the general properties of the fruit before any treatment and transformation (i.e., osmotic treatment). The results obtained from the autumn berries are presented in Table 1.

**Table 1:** Physicochemical properties and antioxidant activity of autumn olive berries (in the present study and from the literature).

Fruit Properties (Present Studies) Mean ± S. D		Fruits Properties (Previous Studies)	Reference
Color parameters: L*	31.53 ± 1.20 ^a^	NF	-
a*	6.10 ± 1.23 ^a^	NF	-
b*	4.43 ± 0.44 ^a^	NF	-
Number of fruits/Weight (50 g of berries)	259 ± 6 ^b^	17.54–18.40 g/100 berries	[6]
		16.41–22.80 g/100 berries	[27]
Length mm	6.19 ± 0.64 ^c^	6.99 ± 0.13 mm	[28]
		7.1–8.7 mm	[27]
Width mm	6.40 ± 0.72 ^c^	3.78–4.28 mm	[6]
		4.8–6.7 mm	[27]
Density (ρ) g/cm^3^	1.038 ± 0.010 ^d^	NF	-
Water activity (a_w_)	0.955 ± 0.007 ^d^	NF	-
Moisture content (M.C%. FW)	77.24 ± 0.41 ^e^	78.49–81.71%	[5]
		71.4 ± 1.8%	[6]
Soluble solids content (SSC: °Brix)	14.80 ± 0.27 ^f^	12.3–15.4	[29]
		9.03–11.76	[6]
		11–17	[30]
pH	3.07 ± 0.03 ^f^	3.1–4.0	[29]
		3.30–3.90	[6]
		4.5 ± 0.1	[5]
Titratable acidity T.A% (malic Acid%)	1.20 ± 0.05 ^g^	0.79–1.29 (TA%) FW	[31]
		2.02–6.88 (Malic. A) mg/100 g FW	[31]
		26.59 ± 1.63 mg/g FW (total acids)	[28]
		2.20–2.94% FW	[6]
		3.1 ± 0.1% FW	[5]
Lycopene mg/100 g FW	20.47 ± 3.18 ^g^	33.6–55.3 mg/100 g FW	[29]
		19.9 ± 3.2 (T. Carotenoids) mg/g DW	[5]
		30.58 to 46.23 mg/100 g FW	[31]
		30–50 mg/100 g FW	[30]
Vitamin C. mg A.A Eq/100 g	7.17 ± 2.27 ^g^	7.65–10.10 mg/100 g FW	[6]
		27.8 ± 1.8 mg/100 g FW	[5]
		13.8–16.9 mg/100 g FW	[27]
Total phenols. mg G.A. Eq/100 g FW	287 ± 40 ^g^	1399–1833 mg/kg FW	[29]
		5.56 mg/g DW	[32]
		168.9–258.1 mg G.A. Eq/100 g FW	[19]
		23.3 ± 2.0 mg/g DW	[5]
		16.3–20.0 mg G.A. Eq/g FW	[33]
		1700 mg Chlorogenic. A. Eq/kg FW	[30]
		190–275 mg GA Eq/100 g FW	[34]
Flavonoids mg-(-) Ep. Eq/100 g FW	25.26 ± 3.78 ^g^	3.6 ± 0.1 mg/g DW	[5]
		1.5–3.8 mg quercetin. Eq/g FW	[33]
DPPH. mMol Trolox. Eq/100 g FW	494 ± 48 ^g^	IC_50_ = 0.13 ± 0.01 mmol of Trolox.Eq/g DW	[32]
		IC50 = 2.42–5.37 mg of FW	[35]
		IC50 = 45.40–49.00 µg/mL	[33]
FRAP. mMol. Trolox. Eq/100 g FW	718 ± 43 ^g^	NF	-
Total anthocyanins. mg cyanidin. Eq/100 g FW	ND	ND	[32]

(-): No reference was found, Eq: Equivalent. NF: Not found in the literature. ND: Not detected. FW: Fresh weight. DW: Dry weight. Superscripts a, b, c, d, e, f, and g are the number of replications 15, 6, 20, 3, 8, 5, and 4, respectively.

The color of the berries was a mixture of brightness (L* = 31.53), redness (a* = 6.10), and yellowness (b* = 4.43). The water activity (a_w_) and density (ρ) were determined as 0.955 and 1.04 g/cm^3^, respectively. The color, density, and water activity of autumn olive berries have not been determined in the literature so far. However, the rest of the expected results are following the previous studies carried out for several ecotypes and genotypes of autumn berries (Table 1). For instance, pH, T.A (Malic acid%), SSC, M.C, length, and width were 3.07, 1.20%, 14.8%, 77.24%, 6.19 mm, and 6.40 mm, respectively.

Likewise, many nutrients and compounds exhibited similar contents to those of the literature. In 100 g of fresh fruits, lycopene, vitamin C (ascorbic acid), TPC, and flavonoids were 20.47 mg, 7.17 mg (AA Eq), 287 mg (GA Eq), and 25.26 mg (Ep. Eq), respectively. Total anthocyanins were not detected either in the current study or in the study carried out by Spínola, Pinto, Llorent-Martínez, and Castilho [32]. It was reported that the phenolic compound contents were similar to those of strawberries and blueberries [34]. Additionally, regarding fruit flavor, astringency has been attributed to the high content of total phenolic compounds [30]. Flavonols appear to be the most abundant compounds, making up 79% of the autumn berries’ phenolic compositions [32]. Lycopene is typically used as a natural pigment [36], while phenolic compounds can be used as bioactive ingredients in foods and cosmetics [37].

According to previous studies (Table 1) and due to the high content of phenolic compounds, carotenoids (lycopene), and vitamin C, the calculation of DPPH revealed interesting values. This confirmed the existence of increased antioxidant activity and the ability to scavenge free radicals. In the present study, DPPH and FRAP, representing the ability to quench and reduce free radicals, were spectrophotometrically measured. They revealed a high antioxidant potential, where DPPH and FRAP gave 494 and 718 mMol Trolox Eq in 100 g of fresh weight, respectively.

Due to the contained phytonutrient and functional compounds, autumn berries are widely believed to decrease the occurrence of many ailments (e.g., myocardial infection) and cancer types (e.g., prostate) [5,27]. The extracts of these berries have proved their inhibitory activity against the transcription factors associated with carcinogenesis, as well as against the proliferation of cancer cells [31]. Besides the antioxidant and cancer inhibitory activities, previous studies have shown that autumn olive berries can exhibit action against diabetes (T2DM) and obesity [32].

In addition to phytonutrients, the existence of oil, various sugars, and minerals make these fruits a real source of nutrients [5,27]. With respect to flavor and taste, organoleptic evaluation of the berries showed interesting acceptability [6]. As such, the possession of such nutritional value and such functional power would induce the consumption and use of these berries, thereby producing novel products and supplement formulations.

### 3.2. Optimization Process

#### 3.2.1. Analysis of the Model

In order to study the effect of OD factors (syrup concentration X_1_, temperature X_2_, and fruit-to-syrup ratio X_3_) and to optimize the dehydration process, the experiment was performed using RSM of Box–Behnken design. The experimental data of different responses (SG, WR, WL, ρ, a_w_, and ΔE), together with experimental points (17 runs), were presented in Table 2. The responses for different combinations showed a wide variation for all mass transfer dependent variables (SG, WR, WL, ρ, and a_w,_) and color change (ΔE). The range values of 1.32–27.02, 12.76–43.62, 14.24–58.48, 1.06–1.23, 0.838–0.973, and 3.10–6.65 were obtained for SG, WR, WL, ρ, a_w_, and ΔE, respectively. The highest WR, WL, and ρ were obtained in run 7 (70% Brix, 70 °C and 1.5:10 ratio), while the highest SG was identified at run 15 (70% Brix, 45 °C and 2:10 ratio), the highest a_w_ at run 8 (30% Brix, 20 °C and 1.5:10), and the highest ΔE at run 13 (50% Brix, 20 °C and 1:10 ratio). The lowest values of WR, WL, and ρ were detected at run 8 (30% Brix, 20 °C and 1.5:10 ratio), while the lowest SG, a_w_, and ΔE were obtained at run 16 (30% Brix, 45 °C and 1:10 ratio), run 7 (70% Brix, 70 °C and 1.5:10 ratio), and run 10 (70% Brix, 45 °C and 1:10 ratio), respectively. These findings showed that high X_1_ and X_2_ (70% Brix and ≥45 °C) had an essential effect on increasing the mass transfer between the fruit and dehydration medium.

Multiple linear regression analysis techniques analyzed second-order quadratic polynomial models for predicting the different responses. Stepwise regression was applied to identify only the significant terms and to remove the non-significant ones (*p* < 0.05) (Table 3), leading to the reduced equations. The adequacy and the fitness of the models were tested by ANOVA, together with regression coefficients and the corresponding determination coefficient (*R*^2^), Adj-*R*^2^, and the lack-of-fit tests (Table 3). Response surface plots were generated for all significant responses as a function of two independent variables. The regression equations that describe the effects of the process variables in terms of uncoded factors on the various responses as a function of syrup concentration (X_1_), temperature (X_2_), and fruit-to-solution ratio (X_3_) were given as follows:

(7) $$\mathrm{SG}=8.79+\;5.61\;X_{1}\;+\;4.82\;X_{2}$$

(8) $$\mathrm{WR}=22.08+\;4.01\;X_{1}\;+\;6.34\;X_{2}$$

(9) $$\mathrm{WL}=30.87+\;9.62\;X_{1}\;+\;11.16\;X_{2}+\;2.34\;X_{3}+\;6.09\;X_{1}X_{2}$$

(10) $$\rho \;=1.11+\;0.037\;X_{1}\;+\;0.047\;X_{2}+\;0.024\;X_{2}^{2}$$

(11) $$a_{w}=0.94-\;0.032\;X_{1}-0.029\;X_{2}-0.029\;X_{1}X_{2}$$

(12) $$\Delta E=4.20\;-\;1.04\;X_{2}-0.70\;\;X_{1}^{2}+1.19\;X_{2}^{2}$$

#### 3.2.2. Effects of Independent Variables on Mass Transfer Responses (WL, WR, and SG)

The results of runs and ANOVA analyses were given in Table 2 and Table 3, respectively. As can be seen from these tables, ANOVA analyses showed that R^2^ for WL was 0.9457, and the adjusted R^2^ was 0.9277. These coefficients were found to be higher than those of WR and SG, which were less than 0.6000. Moreover, the coefficient of variation (CV%) was 9.84%, 21.67%, and 55.66% for WL, WR, and SG, respectively. The lack of fit was not significant for WL (*p* < 0.2414) and SG (*p* < 0.2156). However, the lack of fit of WR was substantial (*p* < 0.0087). The models developed for WL, WR, and SG were significant. Thus, these models can be used to navigate the design space.

**Figure 1:**
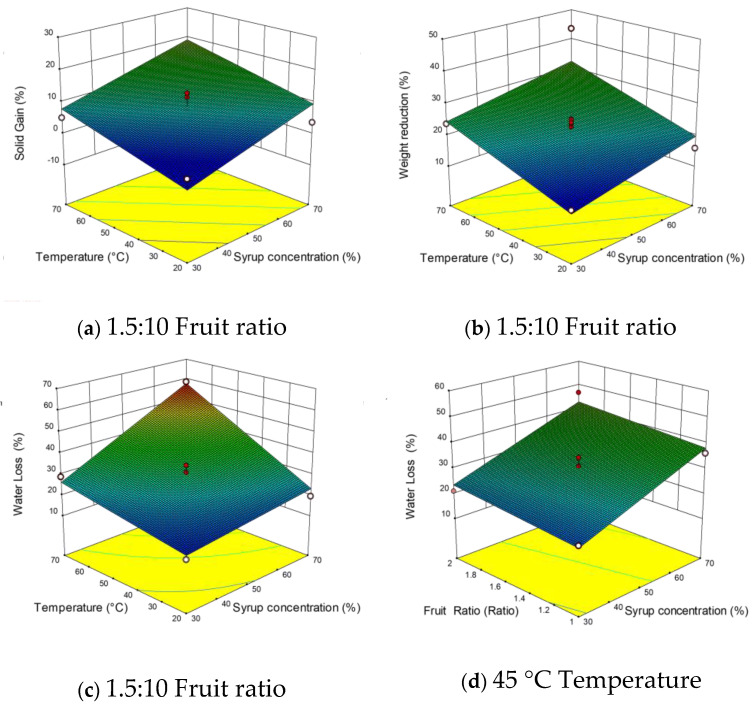

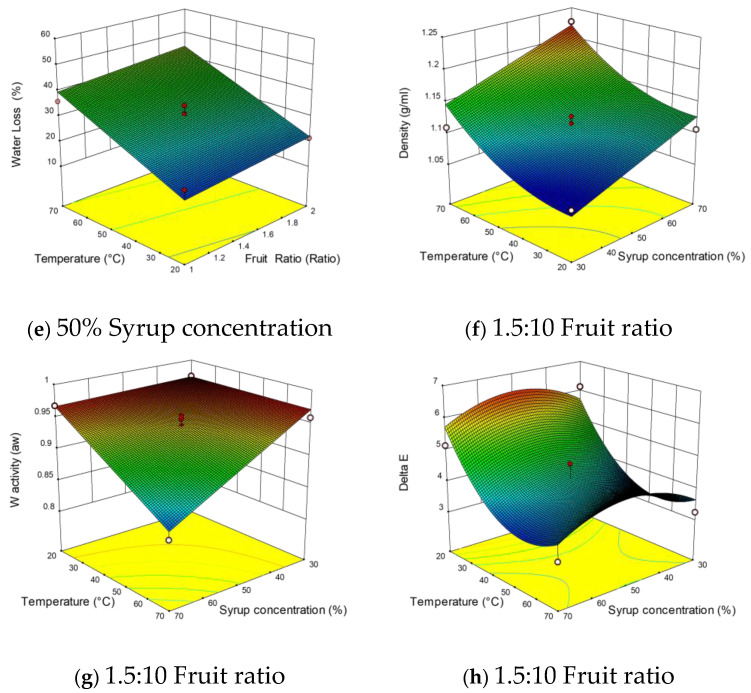
Response surface plots for different mass transfer parameters SG (**a**), WR (**b**), WL (**c**–**e**), ρ (**f**), a_w_ (**g**), ΔE (**h**) during the osmotic dehydration of autumn olive berries.

The linear terms of X_1_ and X_2_ of the WR and SG models were significant, while their quadratic and interaction terms had no significant effect (*p* > 0.05) on these responses. The linear terms X_1_, X_2_, and X_3_ had a considerable effect on WL (*p* < 0.0001). Additionally, only the interaction X_1×2_ had a significant impact on WL. The effects of independent variables on WL, SG, and WR were shown in 3D plots (Figure 1a–e). The response surface showed the variables X_1_ and X_2_ in 3D plots as they highly influenced the responses SG and WR, while kept the variable X_3_ at its central point (1.5:10) as it was indifferent. The increase of variables X_1_ and X_2_ increased SG and WR linearly during osmotic treatment. The highest WR was obtained with the combination of 70% Brix and 70 °C. The highest for SG was identified with the combination of 70% Brix and 45 °C. For the response of SG, the variable X_1_ was found to be more influential than the variable X_2_ with F values of 10.52 and 7.76. For WR, the X_2_ variable was found to be more influential than the X_1_ variable, with F values of 14.04 and 5.62, respectively.

The WL was influenced by all the independent variables investigated in this study. The increase of X_1_ (F value 107.99) and X_2_ (F value 80.32) was found to show a stronger increase in WL, suggesting that they had the most significant effects on this response. These results were following fundamental osmotic treatment theories, where the pressure gradient and driving force for mass transfer are strongly linked to syrup concentration and temperature [38]. However, the increase in the fruit-to-syrup ratio has shown a slight increase in WL. It has been previously shown that the increase of the syrup concentration and temperature had a significant effect on the increase of effective diffusivity for both water and sucrose [38]. In general, the syrup concentration and temperature were reported to positively affect the WL and SG of the osmo-dehydrated fruits. Recently, the highest WL and SG in papaya cubes have been recorded at the highest concentration and temperature (60% Bix, 70 °C), and vice versa, the lowest values at the lowest concentration and temperature (30% Brix, 30 °C) [39].

The mass transfer is influenced by many factors, including temperature, solute concentration and nature, food-to-solution ratio, and food characteristics. The purpose of drying defines the selection of parameters that lead to the desired results (e.g., fruit candying requires a high solid gain besides eliminating water) [9]. A gradual increase of solute concentration rises gradually up the gradient and the driving force between the fruit and the osmotic medium. Furthermore, the application of high temperature accelerates this process. The mass transfer is a temperature-dependent process since a higher temperature has been recognized to promote faster water transfer due to the possible swelling and plasticizing of cell membranes [40] and the decrease of viscosity of the osmotic medium [38]. It was pronounced to find WL percentage higher than SG and WR, since the rate of water removal is much higher than that of the solute penetration, which was favored by the molecular weight difference [9,26]. The increase of syrup-to-fruit ratio over 5:1 has been found to cause a higher rate of WL 9 and mass transfer in amla and apricot [26,41]. However, higher amounts of syrup means more costs for the drying process. Therefore, a lower syrup-to-fruit ratio (solution volume) with a high mass transfer rate is preferred.

#### 3.2.3. Effects of Independent Variables on Density

The density variation might give a clear idea about the mass transfer process. As can be seen in the Table 3, R^2^ was 0.8281, and the adjusted R^2^ was 0.7884. The R^2^ and adjusted R^2^ generated for density were quite close, suggesting conformity between the experimental and predicted data. The coefficient of variation (CV%) was 1.99, and the lack of fit was not significant (0.0643). Thus, the model was adequate for predicting density and navigating the design space. The model showed that the linear terms of X_1_ and X_2_ had positive effects on density by inducing its increase. However, only the quadratic term of X_2_ had a significant impact on the density.

Response surface plots demonstrated the relationship between independent variables (X_1_ and X_2_) and density (Figure 1f). The increases in the two variables have individually increased the density value. The fresh fruits’ (1.04) density rose by the osmosed fruits to reach its peak at 1.23. This increase could be attributed to the penetration of sugar (high density) and water removal. Furthermore, the elimination of entrapped gases due to process conditions and temperature contributed widely to increment the density of the treated berries.

Similarly, in previous studies, the increases in temperature and syrup concentration rose to a certain level of the bulk density of many fruits and vegetables. The osmotic pre-treatment increased the strawberries’ density from 1.08 to 1.29 [42]. The temperature and syrup concentration increased the sugar infusion and the release of air, which promoted water removal and lowered the osmotic medium’s viscosity. Moreover, the modification of the tissue structure and the relaxation of the tissue could play a crucial role in increasing the bulk density [16]. Furthermore, a limited gas phase was retained, inducing volume loss and shrinkage [43,44]. The high bulk density can be desired as a response to the OD process depending on the final product’s further uses.

#### 3.2.4. Effects of Independent Variables on ΔE

The results of ANOVA of the effects of X_1_, X_2_, and X_3_ were given in Table 3. As can be seen, R^2^ was 0.7988, and adjusted R^2^ was 0.7524. The closeness of R^2^ and adjusted R^2^ indicated that there is excellent conformity between the experimental and predicted data of ΔE. CV was determined as 12.69%, and the lack of fit was not significant (0.1928), implying that the model was adequate for predicting the color change and navigating the design space. For this model, only the linear term of X_2_ had significant effects on ΔE, where the decrease of X_2_ increased the color change in autumn olive berries significantly. Meanwhile, both quadratic terms of X_1_ and X_2_ presented significant effects on the color change. X_2_ was the most significant F value 18.78 (*p* < 0.001).

The sharp decrease in ΔE started from 20 °C and reached a valley between 50 and 60 °C before a small increase from 60 to 70 °C (Figure 1h). Although X_1_ was almost indifferent, at 50% concentration, the highest color change occurred. These results were in agreement with previous studies [45]. The color change can be explained by the enzymatic browning reactions that occurred at low temperatures. Additionally, the increase of temperature and syrup concentrations inactivates the oxidative enzymes progressively and increases the solid uptake, reducing oxygen availability [46,47]. Generally, the osmotic pre-treatments helped to reduce the effect of subsequent drying on color (less browning) to obtain food products (apples, grapes) with better characteristics.

#### 3.2.5. Effects of Independent Variables on Water Activity

The results showing the effect of the independent variables on water activity (a_w_) were given in Table 2. For the model developed for water activity (Table 3), R^2^ was found to be 0.8550, adjusted R^2^ was 0.8216, and CV was 1.64%. The lack of fit was not significant (0.0933), indicating that the model was adequate for predicting the water activity and navigating the design space. The linear terms of X_1_ with an F value of 33.55 and X_2_ with a linear F value of 29.16 were found to have significant effects on water activity. The interaction term X_1_X_2_ had a significant effect, while no quadratic terms showed significant water activity effects.

The increase of X_1_ and X_2_ was revealed to positively reduce the water activity (Figure 1g). The combined effects of these variables led to reducing water activity to 0.838 at higher values of 70% Brix and 70 °C. This can be explained on the one hand by the water removal due to the osmosis phenomenon, or on the other hand, by the solute gain and the possible interactions of existing molecular water with solute molecules. It has been previously observed that high solute concentration (salt, sucrose) and temperature have reduced the water activity to less than 0.800 in potatoes. The solute gain is inevitable [48]. Likewise, long osmo-concentration time (12 h) significantly reduced the water activity in blueberries [16]. This supported our results since the experimental time was set to 10 h in this study.

#### 3.2.6. Multi-Response of Optimization

In this study, the parameters investigated ranged from 30 to 70%, from 20 to 70 °C and from 1:10 to 2:10 for syrup concentration (X_1_), temperature (X_2_), and fruit-to-syrup ratio (X_3_), respectively. The second-order polynomial models were used for each response to determine the specified optimum conditions to obtain the desired product. The desirability function was used to optimize the OD process. The criteria consisted of maximizing the water loss and bulk density and minimizing the water activity. Since the dehydrated product is targeted for confectionery, it should be kept for a long storage period by decreasing the water content and increasing the solute uptake. The optimum point was found to be at 70%, 70 °C, and 1.8:10 for solution concentration, temperature, and fruit-to-solution ratio, respectively. Under these optimum conditions, the predicted values were 59.21%. 19.21%, 32.34%, 1.22 g/cm^3^, 0.850, and 3.65 for WL, SG, WR, ρ, a_w_, and ΔE, respectively.

### 3.3. Effects of Drying on Microstructure

**Figure 2:**
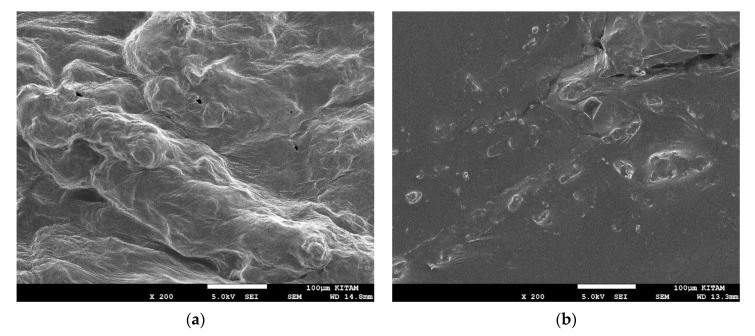
Microstructures of fresh (**a**) and osmo-dehydrated (**b**) autumn olive berries.

The microstructural differences induced by the application of osmotic dehydration were shown by SEM images (Figure 2). The SEM analysis was undertaken on the fresh fruit and the fruit osmo-dehydrated at 70%, 70 °C, and 1.8:10 for solution concentration, temperature, and fruit-to-solution ratio, respectively (optimum conditions). As can be seen, the microstructure of the fresh fruit is a massive amorphous structure with some alveoli-like zones on the surface (Figure 2a). After the osmotic dehydration process, the fruit’s microstructure became smoother, with some cracks on the surface (Figure 2b). This observation denoted that the osmotic dehydration process affected the overall mass transport properties of the tissue. Moreover, the cells shrunk, and some of them collapsed after the water loss. Similar observations have been previously reported for many products [26,49,50].

## 4. Conclusions

After determining the initial nutritional composition and antioxidant activity, autumn olive berries confirmed the importance of their consumption and its transformation into multiple products. The Box–Behnken plan was also used to study the effect of three independent variables (syrup concentration, temperature, and fruit-to-syrup ratio) on the different responses (WL SG, WR, ρ, a_w_, and ΔE) during the osmotic dehydration of these berries. In this research, the concentration and temperature of the syrup were the most effective factors in all responses. However, the fruit-to-syrup ratio factor was only significant on the WL of autumn berries after 10 h of treatment. The regression equations obtained can be used to predict the optimal conditions for the desired product. By applying the desirability function, maximizing the WL and ρ, and minimizing the a_w_, the optimal point was 70%, 70 °C, and 1.8:10 for syrup concentration, temperature, and fruit-to-syrup ratio, respectively. At this point, WL, SG, WR, ρ, a_w_, and ΔE responses were 59.21%, 19.21%, 32.34%, 1.22 g/cm^3^, 0.850, and 3.65, respectively. As a result, autumn berries’ osmotic dehydration can be a good way to reduce the water content and a useful tool before conventional drying (air-drying, freeze-drying). This finding can be helpful for industrial applications. Despite the number of achieved responses, many analyses (texture, shrinkage) and sensorial investigation can be researched to target the final product with desired specifications.

## Figures and Tables

**Table 2:** Uncoded Box–Behnken design independent variables and experimental data of observed responses.

Run	Factors			Responses					
	X_1_	X_2_	X_3_	SG %	WR %	WL %	ρ g/cm^3^	a_w_	ΔE
1	50	45	1.5:10	5.79	25.11	30.91	1.10	0.951	3.95
2	70	20	1.5:10	3.67	15.83	19.50	1.11	0.967	5.14
3	50	45	1.5:10	5.52	23.79	29.32	1.10	0.957	3.75
4	30	45	2:10	2.04	19.02	21.06	1.09	0.967	3.76
5	50	45	1.5:10	6.60	24.12	30.72	1.11	0.955	3.75
6	50	45	1.5:10	12.93	21.43	34.36	1.13	0.937	4.67
7	70	70	1.5:10	14.85	43.62	58.48	1.23	0.838	3.13
8	30	20	1.5:10	1.48	12.76	14.24	1.06	0.973	6.14
9	50	20	2:10	4.75	16.66	21.41	1.09	0.962	6.38
10	70	45	1:10	9.33	26.76	36.08	1.11	0.936	3.10
11	30	70	1.5. 10	5.15	23.70	28.85	1.11	0.958	3.23
12	50	70	2:10	14.58	27.60	42.18	1.21	0.918	4.18
13	50	20	1. 10	4.44	16.54	20.98	1.10	0.961	6.65
14	50	45	1.5:10	11.61	22.71	34.32	1.12	0.941	3.80
15	70	45	2:10	27.02	19.74	46.77	1.19	0.875	4.24
16	30	45	1:10	1.32	18.38	19.70	1.07	0.971	4.00
17	50	70	1:10	18.30	17.59	35.89	1.19	0.914	5.44

X_1:_ Syrup concentration (%), X_2_: Temperature (°C), X_3_: Fruit-to-syrup ratio (1:10–2:10). Responses are the mean of three replications.

**Table 3:** Analysis of variance (ANOVA) for variable responses, and the coefficient predicted by quadratic reduced models.

	SG			WR			WL			ρ			a_w_			ΔE		
	CE	SS	*p*-Value	CE	SS	*p*-Value	CE	SS	*p*-Value	CE	SS	*p*-Value	CE	SS	*p*-Value	CE	SS	*p*-Value
Model	8.79	437.53	0.0029	22.08	450.15	0.0022	30.87	1928.89	<0.0001	1.11	0.031	<0.0001	0.94	0.018	<0.0001	4.2	16.3	<0.0001
X_1_	5.61	251.79	0.0059	4.01	128.75	0.0326	9.62	740.62	<0.0001	0.037	0.011	0.0004	−0.032	7.95 × 10^−3^	<0.0001	-	-	-
X_2_	4.82	185.74	0.0146	6.34	321.4	0.0022	11.16	995.81	<0.0001	0.047	0.018	<0.0001	−0.029	6.91 × 10^−3^	-	1.04	8.66	0.0002
X_3_	-	-	-	-	-	-	2.34	43.98	0.0496	-	-	-	-	-	-	-	-	-
X_1_ X_2_							6.09	148.48	0.0017	-				3.31 × 10^−3^	0.0001	-	-	-
X_1_ X_3_	-	-	-	-	-	-	-	-	-	-	-	-	-	-	-	-	-	-
X_2_ X_3_	-	-	-	-	-	-	-	-	-	-	-	-	-	-	-	-	-	-
X_1_^2^	-	-	-	-	-	-	-	-	-	-	-	-	-	-	-	−0.7	2.08	0.0236
X_2_^2^	-	-	-	-	-	-	-	-	-	0.024	2.46 × 10^−3^	0.045	−0.029	2.46 × 10^−3^	0.045	1.19	5.93	0.0008
X_3_^2^	-	-	-	-	-	-	-	-	-	-	-	-	-	-	-	-	-	-
Residual		335.04			335.04			110.65			6.49 × 10^−3^			3.08 × 10^−3^			4.11	
Lack of Fit		285.91	0.2156		312.54	0.0087		89.68	0.2414		5.98 × 10^−3^	0.3775		2.78 × 10^−3^	0.0933		3.49	0.1928
Total		772.57			770.66			2039.54			0.038			0.021			20.41	
R^2^		0.5663			0.5841			0.9457			0.8281			0.855			0.7988	
Adj-R^2^		0.5044			0.5247			0.9277			0.7884			0.8216			0.7524	
Pred-R^2^		0.3178			0.2747			0.8621			0.6674			0.7374			0.637	
CV%		55.66			21.67			9.84			1.99			1.64			12.69	

The terms and model are significant at *p* < 0.05, lack of fit is not significant at *p* > 0.05.

## Data Availability

Data sharing not applicable.
